# Beyond place cells: Specific and cooperative roles of hippocampal interneuron families in spatial coding

**DOI:** 10.1016/j.fmre.2026.04.010

**Published:** 2026-04-16

**Authors:** Jun-Wei Cao, Changyou Jiang

**Affiliations:** a School of Life and Health Sciences, Hunan University of Science and Technology, Xiangtan 411100, China; b State Key Laboratory of Brain Function and Disorders, MOE Frontiers Center for Brain Science, Institutes of Brain Science, Fudan University, Shanghai 200032, China; c Research Unit of Addiction Memory, Chinese Academy of Medical Sciences (2021RU009), Shanghai 200032, China

How do we find the exact road back to our home? A body of research has shown that the hippocampus, in concert with its partner structures, constructs a cognitive map that enables flexible navigation through both spatial and mental domains [1]. In the prevailing model, the hippocampal cognitive map is instantiated by the collective place fields of individual excitatory pyramidal neurons [2]. Rather than being fixed, the place fields of individual pyramidal neurons exhibit context-dependent plasticity and remapping [3]. However, the mechanisms governing the generation of these place fields, as well as the neural basis for their flexibility or remapping, remained unclear.

Most physiological studies posit that place fields are generated by the recurrent excitatory system of the CA3 region or the entorhinal grid system, while their flexibility or remapping is attributed to plasticity in excitatory synapses [4]. Meanwhile, the activity of excitatory neurons—from individual firing patterns to network-level dynamics—is finely orchestrated through the specific and cooperative actions of diverse interneuron families. Therefore, diverse inhibitory microcircuits are well-positioned to enable this flexibility through the modulation of pyramidal neuron place fields. Indeed, hippocampal interneurons have been found to exhibit place field–like properties, including on and off fields, theta phase precession, remapping, choice prediction, and learning-related plasticity [5,6]. How did the complex interactions among diverse, genetically defined interneuron families collectively regulate various place field properties? Addressing this question requires simultaneous *in vivo* recordings from multiple members of neuronal families. However, this poses a major challenge, as traditional optogenetic approaches are typically constrained to tagging or manipulating only one—or at most two—cell types per experiment [7], thereby precluding the simultaneous observation of interactions across distinct major interneuron families. A recent study by Valero et al. in *Science* has overcome this limitation with a revolutionary methodology that integrates cell-type-specific optogenetic tagging, *in vivo* physiological recording, and machine learning algorithms into a classifier capable of identifying major neuronal families in large-scale, unlabeled recordings [8] (Fig. 1). Aided by this groundbreaking method, the authors revealed the specific and cooperative roles of major interneuron families in hippocampal spatial coding.

**Fig. 1:**
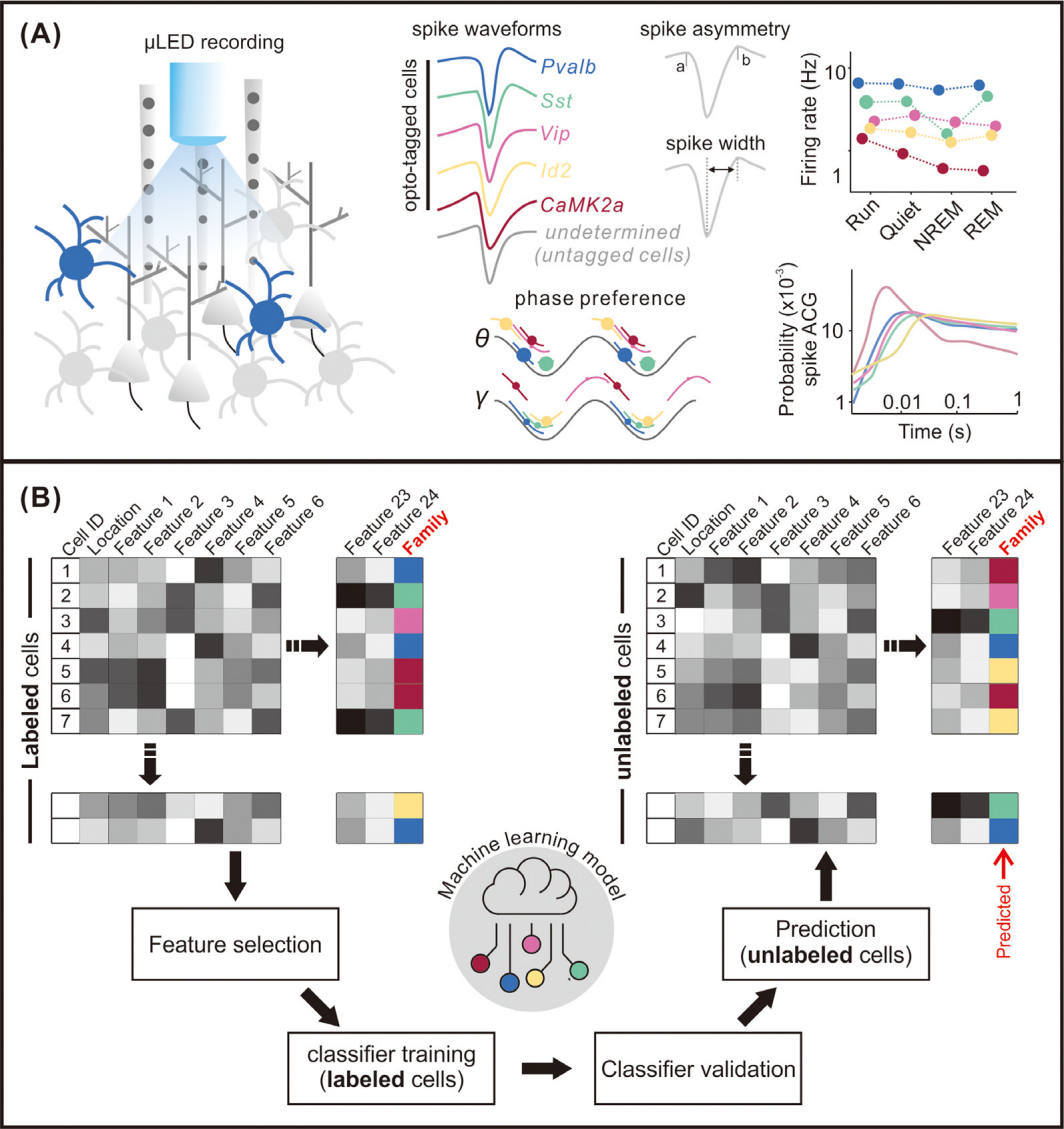
**Supervised classification of hippocampal interneuron families by integrating machine learning and optogenetic recordings.** (A) Specific interneuron families were optogenetically labeled and activated, and their evoked spiking activity was recorded using multi-channel silicon probes. The authors extracted key physiological features—including spike dynamics, firing rates, and phase preference in circuit oscillations—for subsequent classifier training. (B) Using these opto-tagged neurons and their physiological features as a training dataset, the authors trained a high-accuracy decision tree classifier. This trained classifier was then used to predict the family identity of unlabeled neurons based on the same set of physiological features.

To begin with, the authors used optogenetics to individually target specific interneuron families (Pvalb, Sst, Vip, and Id2) and pyramidal neurons (CaMK2α), and then performed *in vivo* physiological recordings. A total of 7532 units were collected from all five opto-tagged neuronal families, of which 785 came from the four interneuron populations. Subsequently, the authors trained decision tree classifiers using physiological features extracted from tagged neurons. The resulting classifier, based on only six features, distinguished the five neuronal families with high accuracy (generally > 89%). Then, the authors applied this classifier to all hippocampal neurons, including numerous unlabeled ones, and obtained five predicted neuronal families. The low-dimensional distribution profile of the five neuronal families derived from physiological features recapitulated that derived from gene expression, and the proportions of these predicted neuronal families were strongly correlated with the gene expression–based fractions (ρ = 0.99, *P* = 0.017), thereby validating the classifier. Classification models powered by advanced machine learning algorithms and large-scale datasets (e.g., single-cell RNA sequencing and *in vivo* electrophysiological recordings) have profoundly transformed the practice of cell type identification [9]. The study by Valero et al. exemplifies this transformation. As the physiological features used for classifier training were obtained from mice in their home cage during spontaneous activity, this physiological fingerprinting strategy is paradigm-independent and thus applicable not only to datasets from different experiments and laboratories but also to investigations of nonspatial hippocampal functions and, potentially, to human electrophysiological recordings.

The authors then sought to elucidate the synaptic and functional coupling patterns among major neuronal families, a prerequisite for mechanistically understanding the functional roles of circuit components. The resulting synaptic response matrix showed that activation of Pvalb, Sst, and Id2 neurons suppressed all neuron groups in a family-specific manner. In addition, a classical disinhibitory microcircuit mediated primarily by Vip neurons (Vip→Sst) was identified, consistent with *in vitro* recordings. Moreover, the authors revealed a strong correlation between the synaptic response matrix and functional coupling matrix (estimated using statistical models) among neuronal families (ρ = 0.73, *P* < 0.001), thereby suggesting that these synaptic connection motifs underlie functional coupling during spatial coding. Overall, these microcircuit motifs provide a foundation for a mechanistic understanding of how specific and cooperative actions among diverse interneuron families and excitatory pyramidal cells shape hippocampal spatial representation. More broadly, it offers a blueprint for understanding hippocampal functions across various behavioral conditions from the perspective of neuronal microcircuits.

Capitalizing on the ability to simultaneously identify multiple neuronal families without a priori genetic labeling, the authors investigated, with pyramidal cells as a reference, the spiking activities of these families during a delayed maze-arm alternation task. While pyramidal neurons, as expected, exhibited classical place fields and decoded the animal’s position with high accuracy, interneurons also showed significant position-dependent rate modulation, with their spiking patterns spanning the full length of the maze. Remarkably, a decoding model based solely on interneurons achieved performance comparable to the pyramidal cell model when neuron numbers were matched between groups. These results demonstrate that hippocampal interneurons, much like classical place cells, directly contribute to spatial coding, rather than merely modulating information flow through local or long-range excitatory circuits.

What are the specific roles of each interneuron family, and how do different families cooperate in spatial coding? First, leave-one-out models—excluding one interneuron family at a time—produced higher decoding errors than models including all families, underscoring the necessity of their cooperative engagement for accurate spatial coding. Second, an analysis of four standard place field features across all interneuron families revealed distinct contributions of each family to position coding. Valero et al. further analyzed how place cells’ features were modulated by the interaction of interneuron families. Results showed that interneuron families play preferential roles in shaping spatial coding features, supporting the concept of a “division of labor” among the various interneuron families. Apart from directly innervating surrounding place cells in a domain-specific manner—such as perisomatic inhibition by Pvalb and distal dendritic targeting by Sst—members of CA1 interneurons also differentially modulate both intrahippocampal (e.g., CA3) and extrahippocampal (e.g., entorhinal cortex) excitatory inputs to CA1 place cells [10]. These intricate interactions between excitatory inputs and local inhibition jointly shape place fields and therefore provide a neural basis for flexible cognitive behavior.

Finally, the authors performed an *in vivo* optogenetic perturbation experiment to directly test the proposed hypothesis that global network cooperativity of interneuron families is critical for spatial coding in pyramidal neurons. Overall, optogenetic activation of distinct interneuron families induced feature-specific changes in the spatial coding of pyramidal place cells. Specifically, Vip activation enhanced place field amplitude, while Sst activation produced the strongest suppression of place field firing rate—a finding consistent with the known disinhibitory Vip-Sst circuit. Moreover, the experiments revealed a prominent time-division control of pyramidal cell place fields, with the first half suppressed by Pvalb activation and the second half by Sst or Id2 activation. These results provide *in vivo* evidence supporting the long-standing hypothesis that distinct interneuron cell types in cortical circuits act in discrete time windows, reflecting a temporal “division of labor” in modulating network activity [11]. This time-division control also provides a mechanistic explanation for the shift in place cell excitation from entorhinal to CA3 input as the animal traverses the place field, whereby sequential potentiation of the place cell-Sst synapses and the feed-forward depression of place cell-Pvalb transmission reveal the coordination and complementarity among interneuron families in shaping place fields.

Methodological breakthroughs and related key findings of the study by Valero et al. have broader transformative implications, ranging from fundamental neuroscience to clinical translation to next-generation artificial intelligence (AI). In fundamental neuroscience, this study establishes a generalizable experimental and analytical framework for causally linking defined cell types to circuit function and behavior across brain regions. Moreover, Valero et al. challenged the long-held view that excitatory neurons are the brain’s primary information encoders, whereas inhibitory neurons are relegated to modulating information flow and coordinating network dynamics. Instead, their findings reveal that interneurons also play an active role in encoding spatial information in the hippocampus, prompting a rebalancing of our understanding of the relative contributions of excitation and inhibition to spatial coding. For clinical translation, the specific interneuron interaction motifs identified in this study—such as Vip→Sst disinhibition—could serve as precise circuit targets for therapeutic intervention in neurological disorders where circuit dysfunction is a central feature, which underscores the importance of this mechanistic understanding for developing targeted neuromodulation or pharmacological strategies. In AI, the demonstration that robust spatial coding emerges from a cooperative, specialized division of labor among diverse interneuron types offers a compelling biological blueprint for improving artificial neural networks, pointing toward more energy-efficient, adaptive, and generalizable AI systems.

Although the physiological fingerprinting strategy developed by Valero et al. enables simultaneous prediction and investigation of multiple neuronal cell types in a single experiment, it also comes with several potential limitations and technical constraints that warrant consideration in practical applications. Because multiple functional subtypes with distinct physiological properties can exist within the same genetically defined family, the current physiological fingerprinting strategy, which relies on prespecified physiological features, is limited in its ability to resolve these subtypes. Therefore, the current classification framework may risk masking finer-scale functional heterogeneity within families. Related to this, the findings derived from this coarse classification framework provide only a broad and preliminary understanding of underlying mechanisms. Successful implementation of this strategy depends on several critical factors, including sufficient recording duration, adequate data volume (especially for rare cell type identification), and rigorous data analysis (e.g., accurate spike sorting). Moreover, since firing patterns are a key feature in physiological fingerprinting, reliably identifying neuronal subtypes with intrinsically low firing rates can be difficult.

In summary, this study describes a novel method using a machine learning classifier based on optogenetically tagged physiological features to classify interneurons *in vivo* and reveal the specific and cooperative roles of inhibitory microcircuits in spatial coding. Further studies will need to identify the physiological fingerprints of the various subgroups within each major interneuron family, and specifically targeting these discrete subtypes will be crucial for a more complete understanding of spatial coding mechanisms.

## CRediT authorship contribution statement

**Jun-Wei Cao:** Writing – review & editing, Writing – original draft, Conceptualization. **Changyou Jiang:** Writing – review & editing, Writing – original draft, Supervision, Conceptualization.

## Declaration of competing interest

The authors declare that they have no conflicts of interest in this work.
